# Immunotherapy of the Dunning Leukaemia with Thymic Extracts

**DOI:** 10.1038/bjc.1973.150

**Published:** 1973-10

**Authors:** Ban An Khaw, A. H. Rule

## Abstract

Injections of thymic extract (TE), TE primed lymphocytes or normal lymph node cells were effective in bringing about total remission of the Dunning leukaemia in inbred Fisher CD rats. Survival greater than 365 days occurred in 10-80% of the various treated groups whereas untreated leukaemic rats, or leukaemic rats treated with spleen “mock thymus extract” or bovine serum albumin, died within an average of 10-17 days. Administration of antilymphocyte serum into leukaemic rats enhanced their death rate. Stimulation of cell mediated immunity *via* the production of functional thymus stimulated lymphocytes is postulated as the mechanism by which tumour rejection occurred.


*Br. J. Cancer* (1973) **28**, 288

### IMMUNOTHERAPY OF THE DUNNING LEUKAEMIA WITH THYMIC EXTRACTS

BAN AN KHAW[1] AND A. H. RULE[2]

*From the Department of Biology, Boston College, Chestnut Hill, Massachusetts*

Received 11 June 1973. Accepted 9 July 1973

**Summary.—Injections of thymic extract (TE), TE primed lymphocytes or normal lymph node cells were effective in bringing about total remission of the Dunning leukaemia in inbred Fisher CD rats. Survival greater than 365 days occurred in 10–80% of the various treated groups whereas untreated leukaemic rats, or leukaemic rats treated with spleen " mock thymus extract " or bovine serum albumin, died within an average of 10–17 days. Administration of antilymphocyte serum into leukaemic rats enhanced their death rate. Stimulation of cell mediated immunity *via* the production of functional thymus stimulated lymphocytes is postulated as the mechanism by which tumour rejection occurred.**

THYMUS mediated immune surveillance has been indicated as an important mechanism in protecting the host from cancers arising from the reticuloendothelial system (Burnet, 1970; Good and Finstad, 1969; Hollinshead, Glew and Bunnag, 1970; Eilber and Morton, 1970). The effectiveness of thymic hormones in stimulating immune recognition and elimination of cancers arising in the reticuloendothelial system is unknown.

In 1966 Goldstein, Slater and White described a dialyzable glycoprotein extract obtained from calf thymus which they labelled thymosin. *In vivo* studies indicated that this thymic extract (Fraction 3) enhanced allograft rejection, restored immunological competence of neonatally thymectomized mice, reduced the incidence of wasting disease and was partially effective in developing resistance to virus induced murine sarcoma tumours (Hardy *et al.*, 1968; Law, Goldstein and White, 1968; Asanuma, Goldstein and White, 1970; Zisblatt *et al.*, 1970).

*In vitro* studies by Bach *et al.* (1971) have indicated that thymosin induces differentiation of primitive cells of bone marrow origin (B cells) into long lived lymphocytes bearing theta antigen (T cells). Incubation of B cells with thymosin has been shown to produce a new population of lymphoid cells with sensitivity to azathioprine, antilymphocyte or antitheta sera.

Miller, Schmiege and Rule (1973) have shown that thymic extracts (TE) could substitute for the presence of T cells in synergistic collaboration with B cells in the humoral antibody response to sheep erythrocytes. These studies indicated that TE induced functional T cell activity.

In this study the same thymic extract was administered to inbred Fisher-CD rats carrying a syngeneic monocytic leukaemia initially discovered by Dunning and Curtis in 1957. Syngeneic lymphoid cells derived from this same line or heterologous antithymocyte serum were also administered to leukaemic rats to investi-

[1] In partial fulfilment of the Master of Science degree.

[2] At present at Mount Sinai School of Medicine of the City University of New York City (on leave of absence). Supported in part by Aid-to-Cancer Research, Boston, Massachusetts.

gate changes in mean survival time in these animals. The purpose of this investigation was to augment rejection or acceleration of the Dunning leukaemia with known modalities and thymic extract to alter thymic-derived lymphocyte immune response.

### MATERIALS AND METHODS

Fresh calf thymus and spleen were obtained from a local slaughterhouse, cleaned, defatted and connective tissue removed. Thymus extract (Fraction 3) prepared according to Goldstein *et al.* (1966) was lyophilized within 24 hours and stored at − 20°C for later use. " Mock " thymic extract was prepared from spleen by the same procedure. Water used in all procedures was first run through 5·0 and 0·45 μm filters, 2 deionizing columns and a charcoal bed. This treatment rendered the water free of ions and bacterial contamination. These preparations did not contain endotoxin, nor conversely could endotoxin substitute for thymic extract in the synergistic collaboration studies.

Inbred leukaemic Fisher-CD rats (150 g) were obtained from the Arthur D. Little Co. of Cambridge, Mass. This company maintains the Dunning leukaemia by 2 methods. One includes the weekly passage of $10^5$ cells in 0·1 ml of saline injected intraperitoneally as the ascites tumour. Cells were also maintained from much earlier passages frozen in 5% glycerine containing Eagle's basic medium and 20% foetal calf serum at − 100°C in the tumour bank. Normally the ascites tumours were passaged in our own laboratory for at least 2–4 passages in Fisher-CD rats whence leukaemic cells were obtained from either current or frozen cell passage.

Fisher-CD rats obtained from the Charles River Breeding Laboratories were fed purina chow and water *ad libitum*. Peritoneal fluids obtained from leukaemic rats were washed 3 times in saline at 1500 rev/min in an international clinical centrifuge. Leukaemic cell counts were determined by the methods described by Todd, Sanford and Wells (1957), and $10^4$ per ml of these cells were transferred to new hosts by intraperitoneal injections in all experimental animal groups.

Intravenous TE therapy was administered to 60 rats (10/group) according to the following schedule: one group received 5 mg/day of buffered TE, pH 7·2, Day −3 to Day 0; another, 5 mg TE/day from Day −3 to Day +4; and the last group 5 mg TE/day from Day +1 to Day +4. The fourth group of leukaemic rats not treated with TE received 0·5 ml of unabsorbed rabbit anti-rat thymocyte serum from Day −3 to Day −1 and 0·25 ml of absorbed rabbit anti-rat thymocyte serum from Day 0 to Day +4. A control group received 5 mg of spleen " mock TE " per day from Day +1 to Day +4. All experimental groups, including the non-treated control group, received $10^4$ leukaemic cells/rat on Day 0. Experiments were repeated twice. Survival rates of rats within each group were determined. These are shown in Table I.

TE primed lymphoid cells were obtained by injecting 1 ml of 15 mg/ml buffered TE/rat intravenously into a group of 9 rats. Each rat received one injection every 48 hours for a total of 3 injections. Twenty-four hours after the last injection the rats were sacrificed. The thymus, spleen and mesenteric lymph nodes from TE treated rats were removed. Untreated rats were also sacrificed and thymus, spleen and mesenteric lymph nodes were similarly obtained. These were washed in saline, cut into small pieces, homogenized gently by hand and the homogenate sieved through cheese cloth before spinning at 2000 rev/min for 10 min in an international clinical centrifuge. The packed cells obtained were made up to a total volume of 5 ml in tris buffer. The concentration of white blood cells was determined as described by Todd *et al.* (1957). Cell concentrations were adjusted to approximately $10^8$ cells/ml for use the same day.

All rats received $10^4$ Dunning leukaemia cells on Day 0. On Day 1 different groups of 10 rats received intravenous injections of $10^8$ TE primed lymph node cells, normal lymph node cells, TE primed spleen cells, normal spleen cells, TE primed thymus cells, normal thymus cells or saline.

Leukaemic rats receiving treatment were considered to have obtained complete remission from cancer if their survival time was 365 days or better, counting from Day 0 of the experiment. Many survived over 2 years. The method of Bancroft (1965) was used whenever possible to assess values and the significance of the results obtained.

TABLE I.—*Survival Rates of Rats Bearing the Dunning Leukaemia: Effect of Intravenous Thymic Extract Therapy*

| Group | No. of rats | No. of experiments | Treatment given to each rat | Mean survival time (days) | P value | % Remission (at 1 year) |
|---|---|---|---|---|---|---|
| 1 | 10 | 5 | None | 15·7 | — | 0 |
| 2 | 10 | 3 | Thymic extract 5 mg/day Day −3 to 0 | — | — | 40 |
| 3 | 10 | 3 | Thymic extract 5 mg/day Day −3 to +4 | — | — | 50 |
| 4 | 10 | 3 | Thymic extract 5 mg/day Day +1 to +4 | — | — | 60 |
| 5 | 10 | 2 | Rabbit anti-rat thymocyte serum* 0·5 ml/day (Day −3 to −1)† and 0·25 ml/day (Day 0 to −4)‡ | 13·2 | 0·04 | 0 |
| 6 | 10 | 2 | Spleen " mock TE " 5 mg/day (Day +1 to +4) | 17·4 | 0·1 | 0 |

* No thymosin administered.

† Unabsorbed rabbit anti-rat thymocyte serum.

‡ Absorbed rabbit anti-rat thymocyte serum.

FIG. 1.—Survival of rats with Dunning leukaemia contrasted with thymosin treated and rabbit anti-rat lymphocyte serum treated leukaemic rats.

### RESULTS

In Table I it is shown that untreated leukaemic control rat groups died within 3 weeks whereas 60% of those receiving TE after the inducing dose of $10^4$ leukaemic cells/rat treatment from Day +1 to Day +4 obtained complete remission. Rats that received treatment from Day −3 to Day +4 had better complete remission rates than those that received TE treatment from Day −3 to Day 0. Rats receiving rabbit anti-rat thymocyte serum were more susceptible to Dunning leukaemia than the leukaemic controls ($P < 0{\cdot}04$).

Injections of either bovine serum albumin or spleen " mock TE " in leukaemic rats produced death rates statistically similar to those for the uninjected leukaemic rats. Young rats under 100 g or

TABLE II.—*Survival Rates of Rats Bearing the Dunning Leukaemia: Effect of Lymphoid Cell Replacement Therapy*

| Group | No. of rats | Treatment 30 hours after $10^4$ leukaemic cells | Mean survival time (days) | *P* | % Remission (one year) |
|---|---|---|---|---|---|
| 1 | 10 | None | 9·6 | — | 0 |
| 2 | 10 | $10^7$ Normal lymph node cells/rat | — | — | 10 |
| 3 | 10 | $10^7$ TE primed lymph node cells rat | — | — | 30 |
| 4 | 10 | $10^8$ Normal spleen cells/rat | 9·6 | 0·7 | 0 |
| 5 | 10 | $10^8$ TE primed spleen cells/rat | 12·5 | 0·1 | 0 |
| 6 | 10 | $10^8$ Normal thymus cells/rat | 10·6 | 0·05 | 0 |
| 7 | 10 | $10^8$ TE primed thymus cells/rat | — | — | 10 |

3 weeks of age were not responsive to TE treatment, nor was the intraperitoneal route as effective as the intravenous one used in this study (unpublished data).

The survival rates of rats receiving lymphoid cell replacement treatment are shown in Table II. Normal lymph node cells increased the resistance of rats bearing the Dunning leukaemia but the effect was somewhat less pronounced than that observed when leukaemic rats received TE primed lymph node cell replacement therapy. Normal spleen cells and normal thymus cells were not as effective as TE treated spleen cells or TE primed thymus cells. However, the results in these groups of rats receiving normal spleen cells, normal thymus cells, TE primed spleen cells and TE primed thymus cells were inconsistent.

### DISCUSSION

Thymic extracts contained in Fraction 3 thymosin have previously been reported by Hardy *et al.* (1968) to enhance cell mediated immunity and allograft rejection. Evidence for increased resistance to Moloney virus induced sarcoma with thymosin treatment was also reported by Zisblatt *et al.* (1970). The present investigation suggests that intravenously injected thymic extract (TE) was able to bring about certain percentages of tumour rejections in rats carrying a lethal leukaemia. The results suggested that optimal effectiveness of TE therapy in rendering complete remission from cancer depended upon the time treatment was started. Leukaemic rats receiving 5 mg TE per rat per day from Day +1 to Day +4 showed the best TE induced tumour remission rate. Further experimentation would be needed to establish these data firmly, however.

Rats that received rabbit anti-rat thymocyte serum (ATS), an immunosuppressive agent (Taub, 1970) had an average survival time of only 13·2 days, compared with a mean survival time of 15·8 days in untreated controls. The mode of action of antithymocyte serum is generally considered to be that of removing or rendering ineffective thymus derived lymphocytes. Enhancement of the death rate in the ATS group suggests that T cell elimination augmented the carcinogenic response. Conversely, tumour rejection in rats receiving lymphocytes, TE stimulated lymphocytes or tumour extract indicates the possibility that rejection was the result of enhanced cell mediated immunity. Studies using this same thymosin batch in synergistic collaboration with B cells suggest that the production of functional T cells enhanced recognition and elimination of the leukaemic cell population (Miller *et al.*, 1973).

Thymus mediated immune surveillance in cancers of the reticuloendothelial system have been shown to be of great importance (Burnet, 1970; Good and Finstad, 1969). Prehn (1971), however, has questioned the importance of immune surveillance in tumours of other tissues and organs. Indeed, the relationship of these findings to human carcinogenesis remains obscure, although the importance of immunological therapy in the treatment of human leukaemia has already been indicated by Mathé *et al.* (1969). Whether or not thymic hormones will be more effective than B.C.G. in human immunotherapy of cancer remains open to investigation.

The importance of thymic enhanced cellular immunity or lymphocyte replacement therapy in the rejection of leukaemia in this study is certainly suggested. Data obtained in this investigation strengthen the concept of the endocrine function of the thymus gland as well as thymus derived lymphocytes in the development of cell mediated immunity.

### REFERENCES

Asanuma, Y., Goldstein, A. L. & White, A. (1970) Reduction in the Incidence of Wasting Disease in Neonatally Thymectomized CBA/W Mice by the Injection of Thymosin. *Endocrinology*, **86**, 600.

Bach, J., Dardenne, M., Goldstein, A., Guha, A. & White, A. (1971) Appearance of T-cell Markers in Bone Marrow Rosette-forming Cells after Incubation with Thymosin, a Thymic Hormone. *Proc. natn. Acad. Sci. U.S.A.*, **68**, 2734.

Bancroft, H. (1965) *Introduction to Biostatistics.* Harper and Row. p. 172.

Burnet, F. M. (1970) *Immunological Surveillance.* Oxford: Pergamon Press.

Dunning, W. F. & Curtis, M. R. (1957) A Transplantable Acute Leukemia in an Inbred Line of Rats. *J. natn. Cancer Inst.*, **19**, No. 5, 845.

Eilber, F. R. & Morton, D. L. (1970) Impaired Immunologic Reactivity and Recurrence Following Cancer Surgery. *Cancer, N.Y.*, **25**, 362.

Goldstein, A. L., Slater, F. D. & White, A. (1966) Preparation, Assay and Partial Purification of Thymic Lymphocytopoietic Factor (Thymosin). *Proc. natn. Acad. Sci. U.S.A.*, **56**, 1010.

Good, R. A. & Finstad, J. (1969) Essential Relationship Between the Lymphoid System, Immunity and Malignancy. *Natn. Cancer Inst. Monogr.*, **31**, 41.

Hardy, M. A., Quint, J., Goldstein, A. L., State, D. & White, A. (1968) Effect of Thymosin and an Antithymosin Serum on Allograft Survival in Mice. *Proc. natn. Acad. Sci. U.S.A.*, 3, 875.

Hollinshead, A., Glew, D. & Bunnag, B. (1970) Skin-reactive Antigen from Intestinal Cancer-cell-membranes and Relationship to Carcinoembryonic Antigens. *Lancet*, i, 1191.

Law, L. W., Goldstein, A. L. & White, A. (1968) Influence of Thymosin on Immunological Competence of Lymphoid Cells from Thymectomized Mice. *Nature, Lond.*, **219**, 1391.

Mathé, G., Amiel, J. L., Schwartzenberg, L., Schneider, M., Cotton, A., Schlumberger, J. R., Hayat, M. & de Vassal, F. (1969) Active Immunotherapy for Acute Lymphoblastic Leukaemia. *Lancet*, i, 697.

Miller, H. C., Schmiege, S. K. & Rule, A. H. (1973) Production of Functional T-cells with Thymosin. *Fedn Proc.*, **32**, 879.

Prehn, R. T. (1971) Perspectives on Oncogenesis: Does Immunity Stimulate or Inhibit Neoplasia? *J. Reticuloendothel. Soc.*, **10**, 1.

Taub, R. N. (1970) Biological Effects of Heterologous Antilymphocyte Serum. *Prog. Allergy*, **14**, 208.

Todd, J. D., Sanford, A. H. & Wells, B. B. (1957) *Clinical Diagnosis by Laboratory Methods.* London: W. B. Saunders and Company. p. 219.

Zisblatt, M., Goldstein, A. L., Lilly, F. & White, A. (1970) Acceleration by Thymosin of the Development and Resistance to Murine Sarcoma Virus-induced in Mice. *Proc. natn. Acad. Sci. U.S.A.*, **4**, 1170.


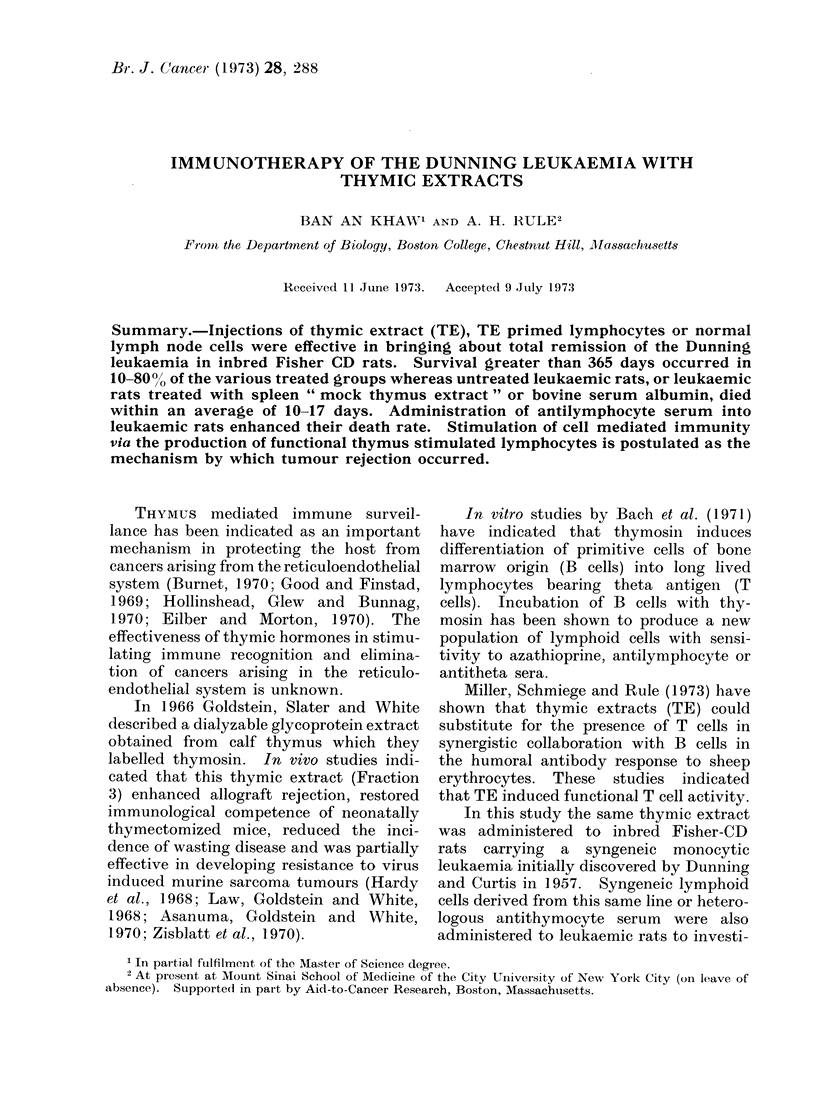

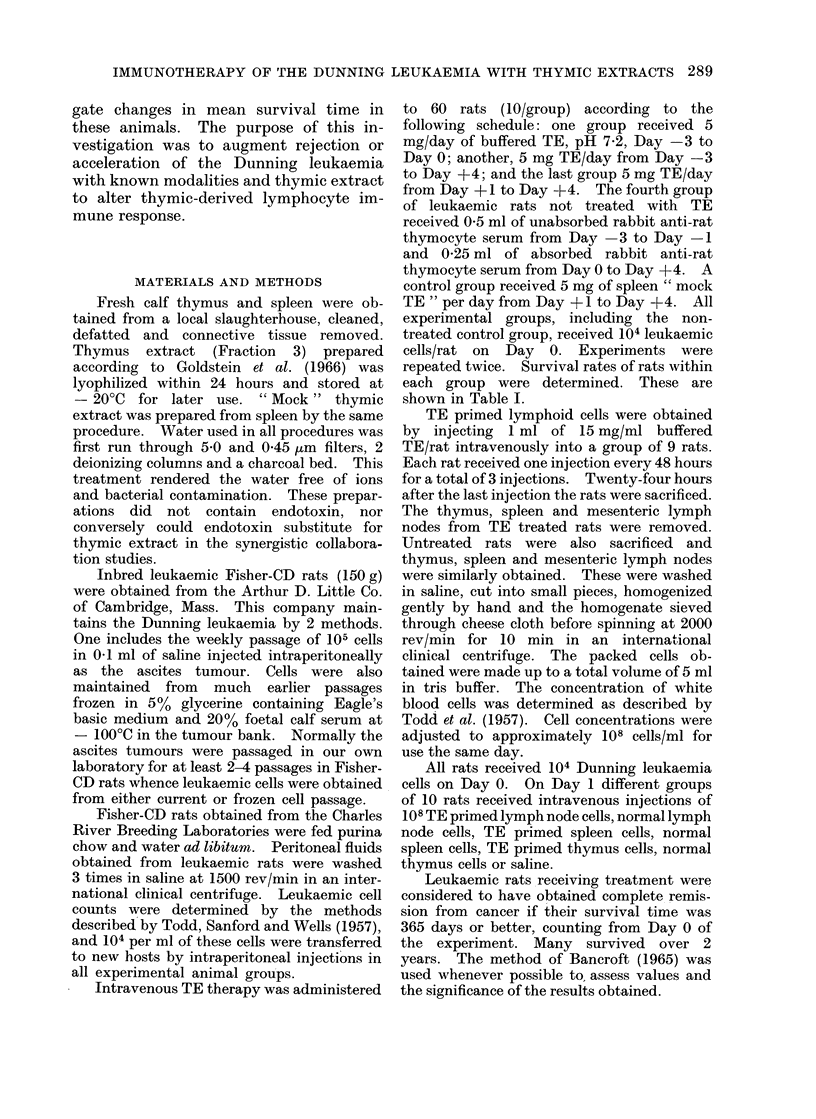

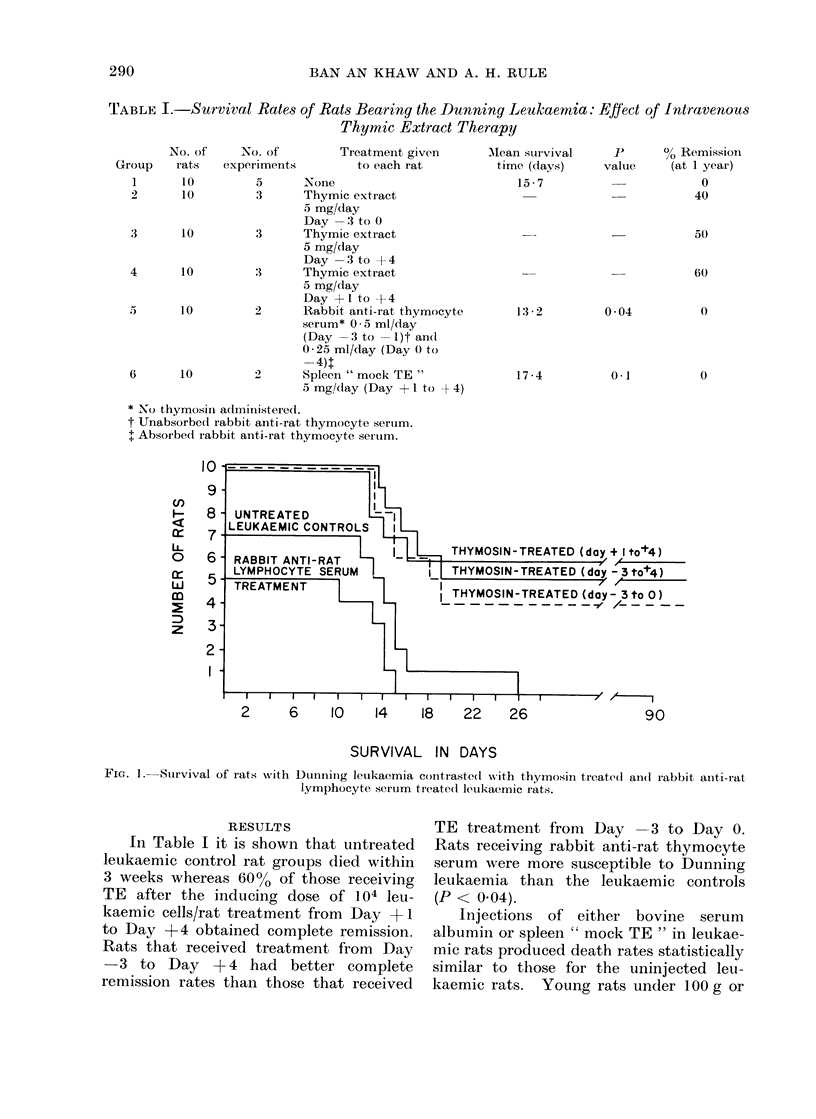

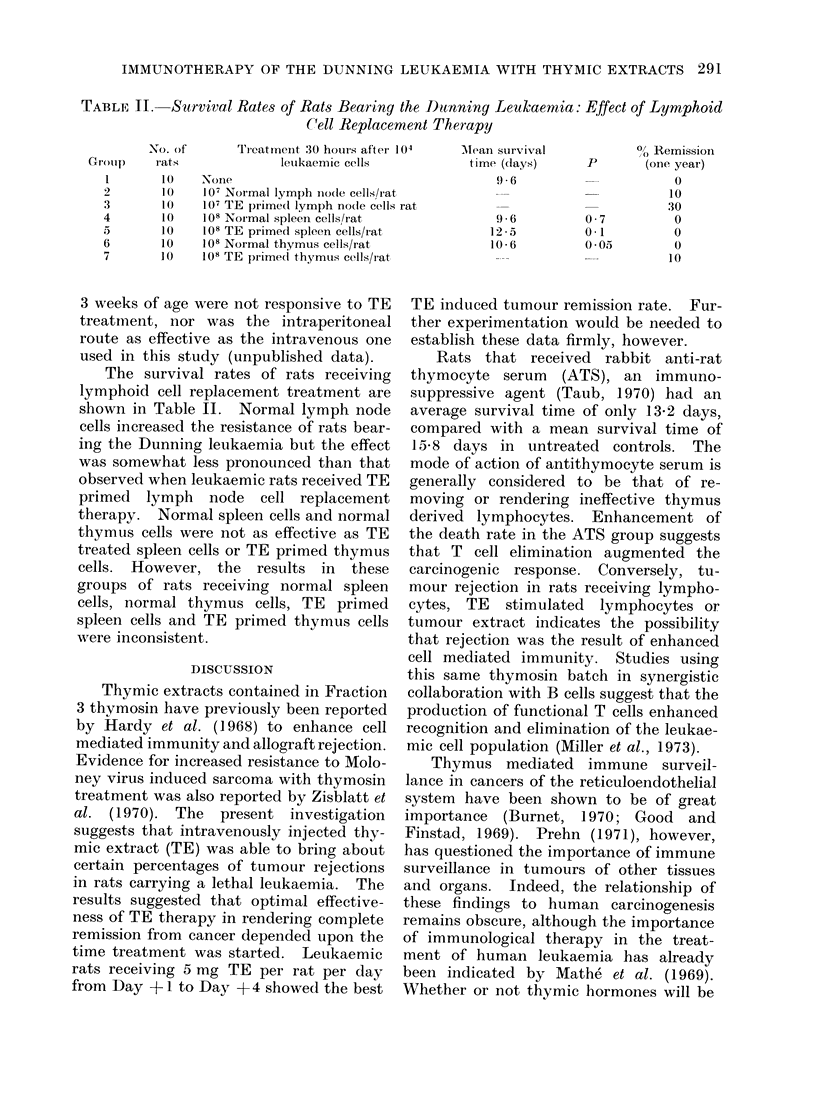

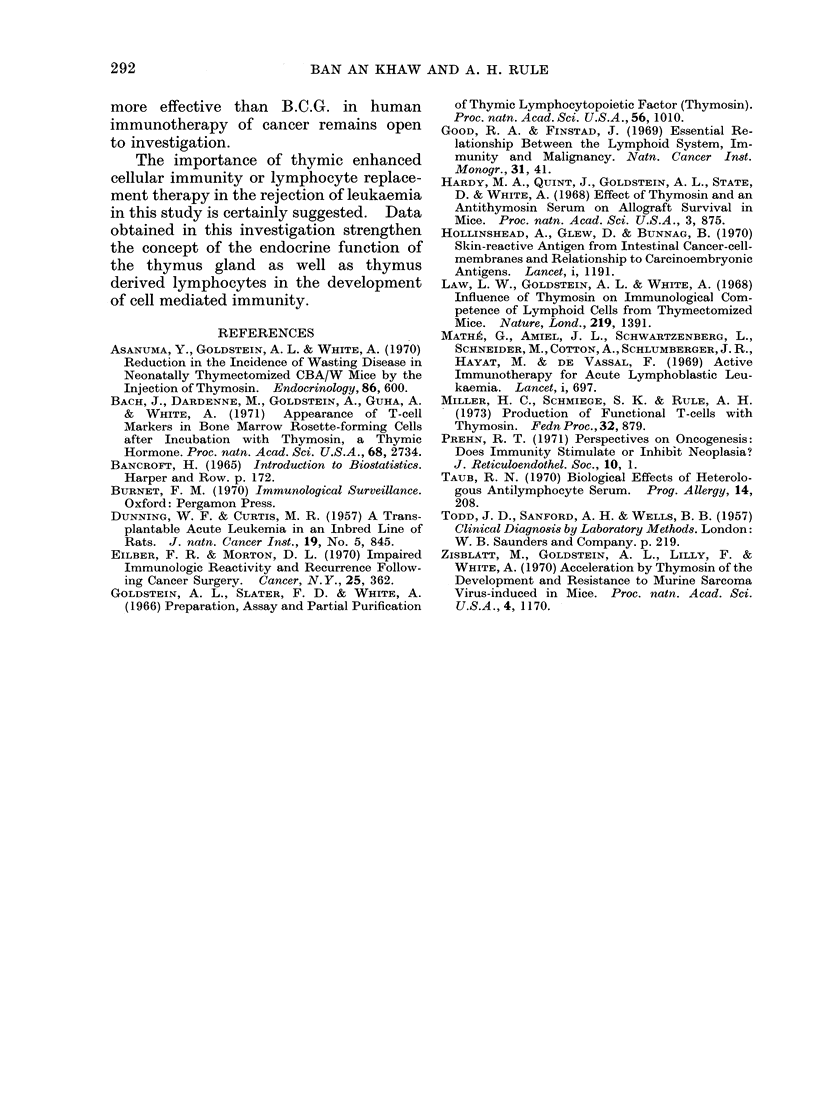


## References

[OCR_00504] Asanuma Y., Goldstein A. L., White A. (1970). Reduction in the incidence of wasting disease in neonatally thymectomized CBA-W mice by the injection of thymosin.. Endocrinology.

[OCR_00510] Bach J. F., Dardenne M., Goldstein A. L., Guha A., White A. (1971). Appearance of T-cell markers in bone marrow rosette-forming cells after incubation with thymosin, a thymic hormone.. Proc Natl Acad Sci U S A.

[OCR_00524] DUNNING W. F., CURTIS M. R. (1957). A transplantable acute leukemia in an inbred line of rats.. J Natl Cancer Inst.

[OCR_00529] Eilber F. R., Morton D. L. (1970). Impaired immunologic reactivity and recurrence following cancer surgery.. Cancer.

[OCR_00534] Goldstein A. L., Slater F. D., White A. (1966). Preparation, assay, and partial purification of a thymic lymphocytopoietic factor (thymosin).. Proc Natl Acad Sci U S A.

[OCR_00541] Good R. A., Finstad J. (1969). Essential relationship between the lymphoid system, immunity, and malignancy.. Natl Cancer Inst Monogr.

[OCR_00547] Hardy M. A., Quint J., Goldstein A. L., State D., White A. (1968). Effect of thymosin and an antithymosin serum on allograft survival in mice.. Proc Natl Acad Sci U S A.

[OCR_00553] Hollinshead A., Glew D., Bunnag B., Gold P., Herberman R. (1970). Skin-reactive soluble antigen from intestinal cancer-cell-membranes and relationship to carcinoembryonic antigens.. Lancet.

[OCR_00559] Law L. W., Goldstein A. L., White A. (1968). Influence of thymosin on immunological competence of lymphoid cells from thymectomized mice.. Nature.

[OCR_00567] Mathé G., Amiel J. L., Schwarzenberg L., Schneider M., Cattan A., Schlumberger J. R., Hayat M., De Vassal F. (1969). Active immunotherapy for acute lymphoblastic leukaemia.. Lancet.

[OCR_00577] Prehn R. T. (1971). Perspectives on oncogenesis: does immunity stimulate or inhibit neoplasia?. J Reticuloendothel Soc.

[OCR_00582] Taub T. N. (1970). Biological effects of heterologous antilymphocyte serum.. Prog Allergy.

[OCR_00592] Zisblatt M., Goldstein A. L., Lilly F., White A. (1970). Acceleration by thymosin of the development of resistance to murine sarcoma virus-induced tumor in mice.. Proc Natl Acad Sci U S A.

